# Orthopoxvirus Infections in Rodents, Nigeria, 2018–2019

**DOI:** 10.3201/eid2902.221411

**Published:** 2023-02

**Authors:** Clement Meseko, Adeyinka Adedeji, Ismaila Shittu, Emmanuel Obishakin, Maurice Nanven, Ladan Suleiman, Daniel Okomah, Visa Tyakaray, Damilola Kolade, Adesola Yinka-Ogunleye, Saleh Muhammad, Clint N. Morgan, Audrey Matheny, Yoshinori Nakazawa, Andrea McCollum, Jeffrey B. Doty

**Affiliations:** National Veterinary Research Institute, Vom, Nigeria (C. Meseko, A. Adedeji, I. Shittu, E. Obishakin, M. Nanven);; Federal Ministry of Agriculture and Rural Development, Abuja, Nigeria (L. Suleiman, D. Okomah);; African Field Epidemiology Network, Abuja (V. Tyakaray);; Nigeria Centre for Diseases Control, Abuja (D. Kolade, A. Yinka-Ogunleye);; US Centers for Disease Control and Prevention, Abuja (S. Muhammad);; US Centers for Disease Control and Prevention, Atlanta, Georgia, USA (C.N. Morgan, A. Matheny, Y. Nakazawa, A. McCollum, J.B. Doty)

**Keywords:** Orthopoxvirus, monkeypox, mpox, rodents, animals, viruses, ecology, ecosystem, One Health, public health

## Abstract

To investigate animal reservoirs of monkeypox virus in Nigeria, we sampled 240 rodents during 2018–2019. Molecular (real-time PCR) and serologic (IgM) evidence indicated orthopoxvirus infections, but presence of monkeypox virus was not confirmed. These results can be used to develop public health interventions to reduce human infection with orthopoxviruses.

Resurgence of zoonotic agent monkeypox virus (MPXV); genus *Orthopoxvirus*) in Nigeria since 2017 calls attention to the need to identify the source of primary transmission at the human–animal interface. Results of previous ecologic investigations of the animal reservoir of MPXV have been inconclusive (*1*,*2*). However, molecular and serologic evidence suggest that potential reservoirs are rope squirrels (*Funisciurus*), sun squirrels (*Heliosciurus*), African pouched rats (*Cricetomys*), and dormice (*Graphiurus*) (*3*–*5*). In Nigeria, investigations into the ecology and natural history of MPXV and its reservoir are limited (*4*). Cases of mpox in humans (formerly monkeypox) in Nigeria were reported in 1971 and 1978 but not again until 2017. During 2017–August 2022, a total of 503 cases of MPXV were confirmed, and evidence of exportation from Nigeria is a public health concern (*6*,*7*). In addition to vaccinating humans, another feasible public health intervention would be identifying and avoiding spillover from animals (*8*). 

As a preliminary step in identifying the putative MPXV animal reservoir in Nigeria, we sampled 240 rodents during 2018–2019. Humane capture and sampling followed protocols approved by the National Veterinary Research Institute and the Centers for Disease Control and Prevention Animal Care and Use Committees (AEC/03/53/18).

We captured and sampled 240 rodents by using a mixture of Tomahawk (https://www.livetrap.com), Sherman (https://www.shermantraps.com), and snap traps in 4 locations: Afi Mountain rain forest, Cross-River state (n = 56); Okomu National Park, Edo state (n = 61); Yenagoa Forest, Bayelsa state (n = 44); and urban and periurban locations associated with recent human cases in Port Harcourt, River state (n = 79). Before collecting blood and euthanizing the rodents, we anesthetized them with inhalant halothane and examined them visually for the presence of poxlike lesions or other signs of illness. If lesions were observed, as seen in animal N198 (Figure), we collected samples from the lesion site. After euthanizing the rodents, we performed necropsies and collected internal organs (e.g., lung, heart, liver, kidney, spleen, skin). For animals not euthanized (as determined for conservation reasons by park officers), we collected only blood and oral and anal swab samples. We extracted DNA by using a MagMAX magnetic processor (ThermoFisher Scientific, https://www.thermofisher.com) and conducted real-time PCR on all available samples by using OPXV-generic and MPXV-specific assays (*9*). We used ELISA to analyze serum and dried blood spots (*5*) and considered samples that were reactive at 1:100 and 1:200 dilutions to be positive.

**Figure:**
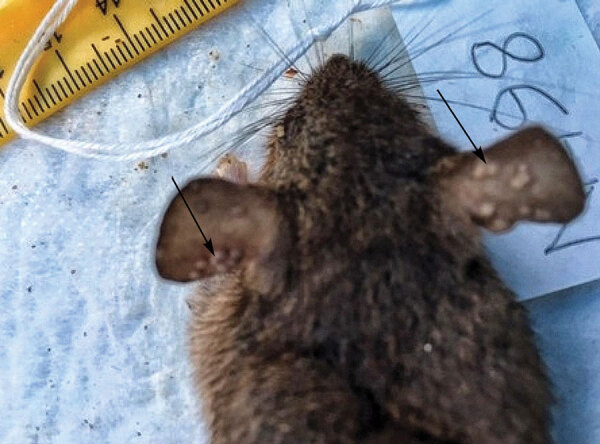
Rodent N198 with poxlike lesions (arrows) in the ears but negative for orthopoxvirus by quantitative PCR and ELISA, sampled in study of orthopoxvirus infections in rodents, Nigeria, 2018–2019.

Animal NG114 (*Mus* *baoulei*), collected from Okomu National Park, Edo, was positive by generic OPXV PCR of skin, spleen, kidney and by dried blood spot but negative for OPXV antibodies by ELISA**.** All samples from that animal were negative by MPXV-specific assay. Samples from all other animals were negative by PCR (OPXV-generic and MPXV-specific). Samples from 2 rodents, NG112 (*Praomys sp*.) and NG173 (*Rattus rattus*), collected in Okomu National Park on December 12, 2018, and from Port Harcourt, River state (prison environment) on July 5, 2019, were positive by ELISA (OPXV IgG). Khodakevich et al. (*4*) detected OPXV antibodies or “MPXV–specific” antibodies in rodents (including *Praomys*) and nonhuman primates in areas where human cases had been reported. A similar investigation in the Democratic Republic of the Congo, conducted in 2012, 2013, and 2015, detected OPXV IgG in 6 rodents, but no rodent was positive by PCR (*9*). Identifying an unknown OPXV via molecular diagnostics (PCR) from a *M.* *baoulei* mouse (NG114) in Okomu and confirmation of ELISA-positive rodents from both urban and rural sites (Port Harcourt and Okomu) indicate the potential role of these animals in the circulation and transmission of OPXVs. The IgG-positive *R. rattus* rat captured at a prison in Port Harcourt indicates potential OPXV exposure of prisoners and of the local population in urban areas through inconspicuous contact with peridomestic rodents.

Our laboratory analyses showed molecular and serologic evidence of OPXV infections (Table); however, they did not confirm presence of MPXV in the animal samples. Because the ELISA cannot distinguish between different species of OPXVs, we could not determine if the 2 animals with OPXV IgG (NG112 and NG173) were exposed to MPXV or another OPXV circulating in small mammals in Nigeria. Detection of diverse OPXVs in rodents in Nigeria can be used to develop public health interventions to reduce human infection with orthopoxviruses, including MPXV. 

**Table:** Samples positive for OPXV in study of OPXV infections in rodents, Nigeria, 2018–2019*

Rodent species	Animal no.	Sample type	OPXV DNA	OPXV IgG
*Mus baoulei*	NG114	Skin biopsy	+	Not applicable
*M. baoulei*	NG114	Spleen and kidney	+	Not applicable
*M. baoulei*	NG114	Dried blood spots	+	–
*Praomys*	NG112	Tissues, dried blood spots	–	+
*Rattus rattus*	NG173	Tissues, dried blood spots	–	+


*By quantitative PCR and ELISA. OPXV, orthopoxvirus.
